# Ultrasound Control of Gene Expression in Human iPSCs via Heat Shock Promoters

**DOI:** 10.1002/bit.70050

**Published:** 2025-08-20

**Authors:** Alessandro R. Howells, Kama Bell, Hyeonu Heo, Tahir Haideri, Yun Jing, Xiaojun Lance Lian

**Affiliations:** ^1^ Department of Biomedical Engineering Pennsylvania State University State College Pennsylvania USA; ^2^ Graduate Program in Acoustics Pennsylvania State University State College Pennsylvania USA; ^3^ Department of Biology Pennsylvania State University State College Pennsylvania USA; ^4^ The Huck Institutes of the Life Sciences Pennsylvania State University State College Pennsylvania USA

**Keywords:** focused ultrasound, heat shock promoter, induced pluripotent stem cells, sonogenetics, synthetic biology, thermal control

## Abstract

Inducible systems are crucial tools in biomedical research, offering researchers spatiotemporal control at the cellular level. A promising development in this field is the use of focused ultrasound for controlling gene expression using heat shock promoters (HSPs). Focused ultrasound‐induced mild hyperthermia activates the cellular heat shock response, which in turn activates HSPs and subsequently drives gene expression. Here, we utilized a Cre/LoxP system where each HSP drives Cre expression to investigate inducible gene expression with HSPs. Cre‐mediated excision at the AAVS1 knock‐in cassette results in constitutive expression of GFP. We assessed the performance of six HSPs in human induced pluripotent stem cells (hiPSCs). HSP16F and synHSPB′3 were the most effective, showing 27.7% and 33.5% GFP positivity, respectively, following 1 h of pulsed 42°C incubations. This contrasts with 0.6% and 3.5% GFP positivity at 37°C, indicating 45.9‐ and 9.7‐fold increases, respectively. Increasing the number of HSP‐Cre transposons did not significantly affect HSP16F but did enhance synHSPB′3, demonstrating its tunability. We then applied focused ultrasound to elevate the temperature to 42°C, resulting in 18.6% and 45.6% GFP positivity for HSP16F and synHSPB′3, respectively, compared to 0.3% and 6.2% at 37°C. Our design requires only a single, brief heat shock treatment to achieve permanent gene expression, enhancing its safety and feasibility for in vivo applications.

## 1 Introduction

A current essential tool of cutting edge biomedical research involves the use of inducible systems (Kallunki et al. 2019). These systems are utilized extensively in the fields of stem cell biology, tissue engineering, functional genomics, gene therapies, neuroscience, and many more (Ma et al. 2016; Chen et al. 2015; González et al. 2014; Reid et al. 2018; Kim et al. 2017; Yesbolatova et al. 2020). Inducible systems endow researchers the ability to spatiotemporally activate or suppress genes of interest (GOIs) by a specific stimulus. The utility and efficiency of these systems are characterized by low leakage during the “off” state (i.e., when the stimulus is absent) and high induction during the “on” state (i.e., when the stimulus is present). If a system is prone to leakage or exhibits low induction, its usefulness is significantly reduced.

A multitude of efficient inducible systems have been developed, most utilizing a small molecule or light to function as the stimulus (Randolph et al. 2017; Gangopadhyay et al. 2019; T. Das et al. 2016; Bijlani et al. 2018; Repina et al. 2017; Müller and Weber 2013; Paoletti et al. 2019; Klippenstein et al. 2018; Emiliani et al. 2022). Generally, this stimulus confers whether a critical upstream protein of that particular circuit is functional. Although these systems operate efficiently in vitro, where the stimulus can evenly diffuse to all cells in a 2D culture, they are less suitable for clinical translation and in vivo applications. This is due to limitations of effectively delivering the stimuli used to the in situ engrafted tissue. Small molecules could be injected intravenously or locally; however, systemic side effects and achieving sufficient mass transport of the drug to the target cells may pose significant challenges (Stieger et al. 2009). Light, on the other hand, does not effectively penetrate through tissue very far (Ash et al. 2017). It therefore often requires surgically mounting a laser close to the target cells (Oh et al. 2021). For both small molecule and optogenetic systems, these limitations are even further magnified when targeting the brain for treatment of neurological disorders. The skull and the blood brain barrier impose difficult obstacles for the stimuli to surpass. Although both systems are effective for in vitro studies, they become invasive and problematic for translational regenerative medicine.

Focused ultrasound (FUS) is a therapeutic technology which can effectively deliver both mechanical and thermal energy non‐invasively up to several centimeters deep into tissue. This results in biological outcomes determined by the tissue type and the acoustic parameters used. By utilizing a range of frequencies, intensities, and continuous or pulsed waveforms, an array of bioeffects can be achieved for therapeutic effect (Meng et al. 2020; Bachu et al. 2021). FUS has been of specific interest for use in the brain as it is capable of penetrating the skull non‐invasively and can be used to reversibly increase blood brain barrier permeability for drug delivery (Burgess et al. 2016). FDA approval has also been granted to treat Parkinson's Disease essential tremor and dyskinesia with transcranial FUS lesioning (Eisenberg et al. 2021). Innovations in transducer arrays and magnetic resonance guidance systems have improved the precision of FUS targeting and allowed for real‐time pressure feedback and temperature monitoring.

The thermal dose delivered from FUS is applied based on the threshold for tissue necrosis. This threshold is dependent on the type of tissue, the degree of vascularization, and the FUS frequency utilized. At high temperatures, cell membranes succumb to protein denaturation, and the tissue undergoes coagulative necrosis within seconds. Continuous‐wave, high‐intensity FUS is used to achieve such thermal ablations. The rapid temperature increase within the focal region can effectively kill tumor cells and create localized lesions. At low temperatures, homeostatic mechanisms are engaged to offset the temperature rise in tissue and hyperthermia can be applied for several minutes to hours, until reaching the threshold for tissue damage. Mild FUS hyperthermia is shown to induce numerous reversible bioeffects, such as increased proliferation, vasodilation, immune response modulation, and increased drug delivery. By pulsing the waveform, the thermal dose applied by FUS can be greatly lowered and precisely controlled, with temperature increase capable of being limited within 1°C.

FUS stimulation has been further explored to non‐invasively engage thermal or mechanoreceptive cellular mechanisms for the purpose of neuromodulation. Low‐intensity pulsed ultrasound waves have been shown to excite or inhibit neurons (Tyler et al. 2008) by mechanically stimulating membrane bound, endogenous mechanosensitive ion channels (Vasan et al. 2022). Inspired by optogenetics (Xu et al. 2023), a new technology dubbed sonogenetics utilizes bioengineering to exogenously express mechanosensitive or thermosensitive ion channels to increase or endow cellular sensitivity to FUS (Ibsen et al. 2015; Bell et al. 2023; Liu et al. 2022). This increased sensitivity allows acoustic intensities to be lowered, effectively increasing the safety of FUS stimulation by utilizing cell‐specific targeting, limiting the chances of undesired biological effects. Utilizing sonogenetic methods, non‐destructive FUS hyperthermia was shown to enable the activation of thermosensitive ligand‐gated ion channels overexpressed in sensory neurons, initiating pain response and modulating neurotransmitter release (Meza et al. 2022). The high spatio‐temporal precision of FUS hyperthermia and the possibility of genetically engineered targeting, as presented through sonogenetics, identify FUS as an ideal stimulus for successful translation of an inducible gene expression system to in vivo applications.

Recently, researchers have shown that slight hyperthermia using thermal energy delivered by FUS can induce exogenous gene expression. This is done by leveraging the endogenous cellular heat shock response, attuned to trigger rapidly after a cell has been put into an environment higher than 37°C (Alagar Boopathy et al. 2022; Velichko et al. 2013). The canonical heat shock pathway involves the liberation of sequestered heat shock transcription factors that trimerize and translocate from the cytosol into the nucleus. There, they bind to specific genomic motifs, called heat shock elements, which in turn change the epigenomic environment so that heat shock related effector genes start getting expressed to negate heat shock (Jaeger et al. 2014; Pessa et al. 2024).

Researchers have utilized this pathway to induce transgene expression after heat shocking cells that have been genetically modified. This modification encompasses genomically integrating a DNA cassette comprised of a heat shock promoter (HSP) upstream of the transgenic GOIs. Various HSPs have been reported, but generally they consist of repeats of heat shock elements upstream of a core promoter. In this way, during heat shock, the heat shock transcription factors bind to both the endogenous and exogenous heat shock elements, driving both their respective expressions.

In a recent publication, several natural and synthetic HSPs were characterized for thermal actuation of different genetic circuits in primary human T‐cells. These cells were heat shocked within a thermocycler at temperatures ranging from 37°C to 42°C (Abedi et al. 2020). In another related paper, the authors developed their own HSPs, containing differing amounts of heat shock elements. They found that seven repeats resulted in the highest expression after heat shocking, with minimal activation with the 37°C controls (Miller et al. 2021). They next showed that their HSP engineered CAR‐T cells enhanced anti‐tumor activity after specifically heating the tumor via photothermic energy. While both studies demonstrated the feasibility of using HSP gene‐expression systems to spatiotemporally control T‐cell activity for CAR‐T immunotherapy, neither utilized FUS as the source of heat shock. One of the first reports for using FUS to actuate HSPs utilized the HSP70 promoter upstream of GFP within C6 glioma cells (Guilhon et al. 2003). They showed that well defined hyperthermia using MRI guided FUS resulted in tight spatial and temporal control of GFP within their in vivo tumor model. Finally, a more recent report demonstrated that FUS could actuate CRISPR systems within engineered human embryonic kidney cells and within T‐Cells, both in vitro and in vivo (Liu et al. 2023).

Although still in its early stages, FUS‐based inducible systems show great promise. However, the optimal temperature thresholds for HSPs can vary depending on the application and cell type (Abedi et al. 2020; Rome et al. 2005), necessitating their careful characterization. Here, we present an in vitro characterization of HSPs in hiPSCs using an HSP‐Cre/LoxP circuitry. We choose to study hiPSCs because they have the potential to differentiate into all cell types in our body (Bao et al. 2016b; Lian et al. 2013, 2012, 2014, 2015; Bao 2016a; Jiang et al. 2021). We applied heat shock either through non‐ultrasound high‐temperature incubation or FUS. This study provides a benchmark for future research on FUS‐mediated gene expression in stem cell‐based regenerative medicine.

## 2 Results

### 2.1 Design Strategy of the HSP‐Cre Heat Shock Inducible System

**Figure 1:**
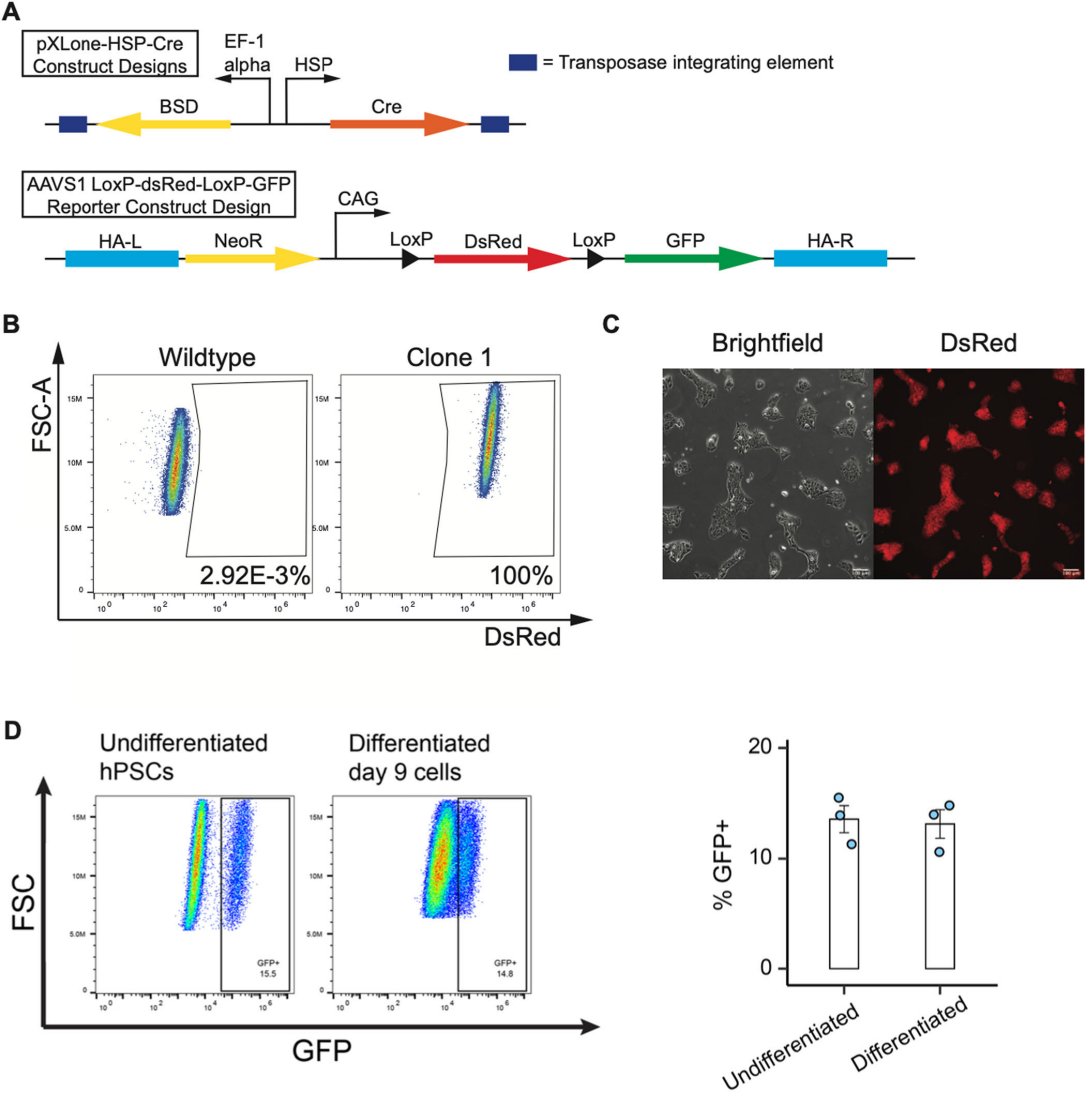
HSP‐Cre/AAVS1‐LoxP‐DsRed‐LoxP‐GFP Clonal hiPSC development. (A) Plasmid DNA cassette construct designs. Top design illustrates the transposase integrating HSP‐Cre construct which also possesses the Bsd drug resistant gene. Bottom design illustrates the AAVS1 safe harbor, CRISPR‐Cas9 knock in donor template, where the constitutive CAG promoter drives DsRed expression. DsRed contains a stop codon and is flanked by two LoxP sequences. Cre recognizes the LoxP sequences and excises DsRed, which subsequently results in the expression of GFP. (B) Live cell flow cytometry of wild type IMR90 hiPSCs and our single cell derived clonal line knocked in with the AAVS1 LoxP‐DsRed‐LoxP‐GFP reporter. (C) Brightfield and fluorescent images of our AAVS1‐LoxP‐DsRed‐LoxP‐GFP reporter clonal line. (D) AAVS1‐LoxP‐DsRed‐LoxP‐GFP hiPSCs were treated with Cre modified mRNA to initiate GFP expression. Then these cells were subjected to neural differentiation for 9 days in a neural differentiation medium (essential 6). Flow cytometry was performed for GFP expression before and after neural differentiation. Scale bars represent 100 µm.

Given that HSP's performance varies across different cell types (Abedi et al. 2020; Rome et al. 2005), we aimed at characterizing HSPs within an optimized manner ideal for its eventual translation to hiPSC based therapies. Given this context, an HSP‐Cre/LoxP circuit strategy was designed, in which a single, relatively short thermal dose of heat shock would result in the permanent and constitutive expression of a GOI (Figure 1A). For simplicity and high throughput characterizations, we opted to use green fluorescent protein (GFP) as our GOI.

First, we knocked the LoxP‐DsRed‐STOP‐LoxP‐GFP reporter construct into the AAVS1 safe harbor site within the IMR90 hiPSC line via CRISPR‐Cas9 technology (Mali et al. 2013). After drug selection, five single‐cell‐derived clones were picked. Clone 1 was chosen for further experimentation because it demonstrated the highest DsRed positivity and mean fluorescent intensity (Figure 1B,C). To determine whether GFP expression in this knock‐in line is transient or stable following Cre activation, we transfected the cells with Cre modified mRNA to initiate GFP expression. The cells were then subjected to a 9‐day differentiation protocol. GFP‐positive cells were quantified by flow cytometry at the end of the differentiation period. Our results showed that the percentage of GFP⁺ cells remained comparable to that observed in undifferentiated hiPSCs, indicating that GFP expression is stable and not transient (Figure 1D).

Next, six candidate HSPs were chosen (Abedi et al. 2020) and cloned into the PiggyBac vector XLone (Randolph et al. 2017). In this construct design, each HSP drives the expression of Cre and a separate EF1a promoter drives the constitutive expression of a blasticidin (Bsd) resistant gene (Figure 1A). Thus, Bsd can be used to drug select hiPSCs that successfully integrated our HSP‐Cre cassettes. The six candidate HSPs (Abedi et al. 2020) were HSP16F, HSPB, HSPmin, HSPB′1, synHSPB′2, and synHSPB′3.

### 2.2 Characterization of Six HSPs Within hiPSCs via Non‐Ultrasound‐Induced Heating

**Figure 2:**
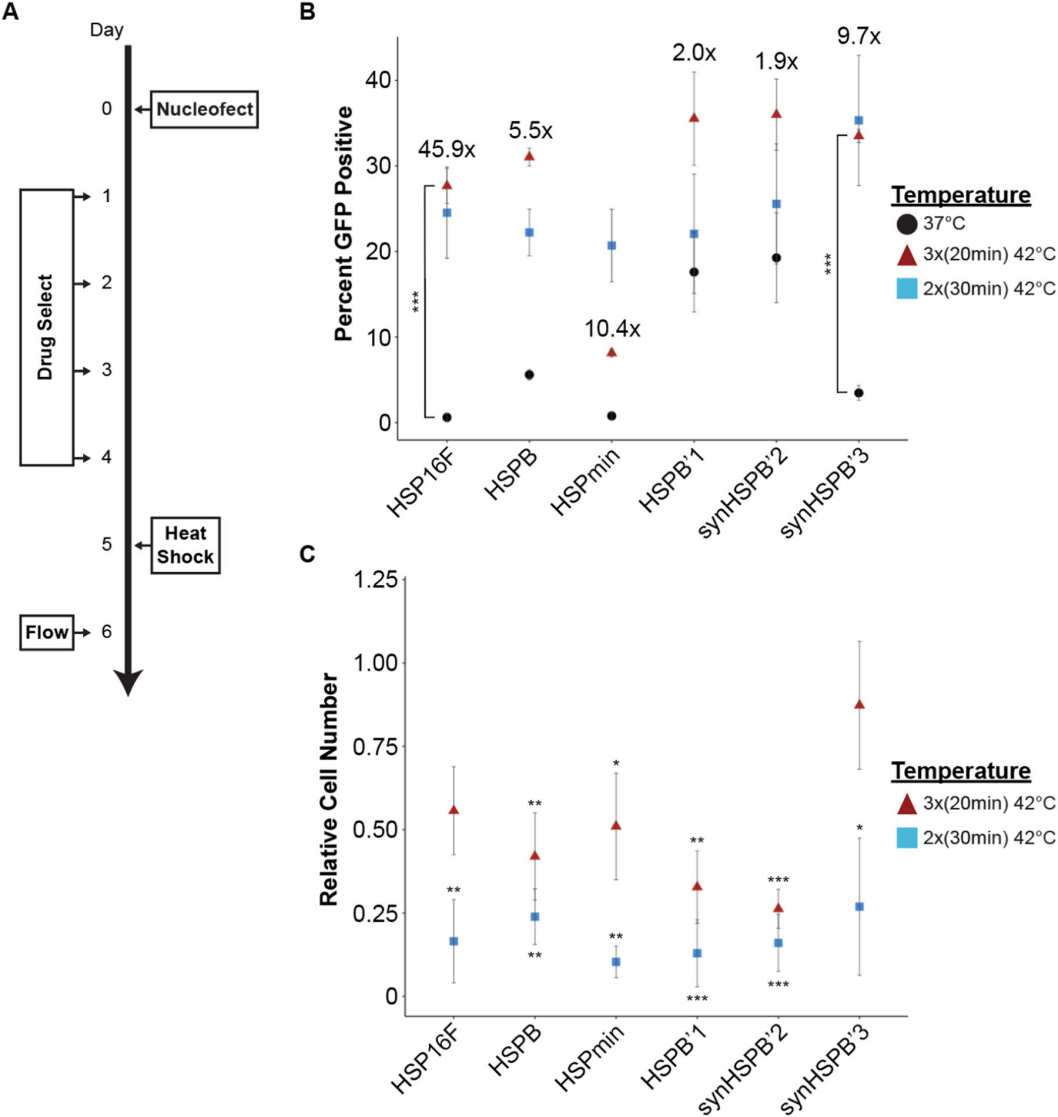
HSP characterization via non‐ultrasound‐induced heating. (A) Schematic depicting experimental design for HSP characterization within hiPSCs. (B) Percent GFP positivity across the six candidate HSPs and across each heat shock parameter. Points depict averages and bars depict standard error (*n* = 3, one‐way ANOVA with post hoc Tukey test ***p < 0.001). Fold change increase shown is calculated using the averages from the 3× (20 min) group and the 37°C control group. (C) Relative cell viability compared to the 37°C control groups. Points depict average and bars depict standard error (*n* = 3, one‐way ANOVA with post hoc Tukey test *p < 0.05, **p < 0.01, ***p < 0.001).

Utilizing our clonal Cre reporter line, we first characterized the performance of these six HSPs to identify the most effective one. HSP performance was assessed in triplicate, by comparing leaky activation (cells kept at 37°C) versus the percent of cells that were activated because of heat shocking. On Day 0, IMR90 AAVS1‐LoxP‐DsRed‐LoxP‐GFP Clone 1 cells were nucleofected with 1000 ng of the HSP‐Cre plasmid along with 1000 ng of a hyperactive transposase plasmid. Cells were nulceofected with a relatively low amount of plasmid to minimize the amount of 37°C leaky activation. Between Days 1 and 4, cells were selected with Bsd treatment. On Day 5, transgenic cells were ready for experimentation. Control cells were left in the 37°C incubator, while experimental cells were non‐ultrasonically heat shocked with either three 20‐min or two 30‐min pulsed incubations at 42°C (i.e., placed in a second incubator set to 42°C followed by an equal time interval in the 37°C incubator, totaling 1 h of 42°C heat exposure). The following day, cells were assessed for Cre expression via live cell flow cytometry to measure the percentage of cells that were GFP positive (Figure 2A).

Our results showed that synHSPB′3 and HSP16F performed the best. They both showed minimal 37°C activation, with an average of 3.5% and 0.6% GFP positivity, respectively. Additionally, they showed 33.5% and 27.7% GFP positivity after heat shocking with the three 20‐min pulses, a significant difference compared to their 37°C counterparts. This translated to a ~10‐fold increase and ~46‐fold increase in activation, respectively (Figure 2B). Across all six HSPs, the three 20‐min pulsed 42°C heat shocking resulted in the highest activation, except for HSPmin, where the two 30‐min pulsed heat shocking resulted in higher activation. Although the two 30‐min heat shocking yielded higher activation in this case, our data illustrated that the two 30‐min condition delivered a thermal dose that was highly toxic, based on our cell viability assay (Figure 2C). Relative to the 37°C control, ~80% of the cells died because of this heat shocking pattern (two 30‐min), while only ~50% of the cells died with the three 20‐min heat shocking. Among six HSPs, synHSPB′3 cells showed a much higher resistance to thermal toxicity. Based on these results, a maximum of 20‐min was chosen to ensure activation while maintaining acceptable cell viabilities, as supported by recommended thermal dose indices for live tissue (Dewhirst et al. 2003).

### 2.3 Comparison of One 20‐min and Two 20‐min Non‐Ultrasound‐Induced Heating

Given the good performance of synHSPB′3 and HSP16F, we further characterized their function using our IMR90 AAVS1‐LoxP‐DsRed‐LoxP‐GFP Clone 1 line for the remainder of the study. First, we examined whether a shorter heat shock could achieve high GFP expression while minimizing cell toxicity. To do this, we heat shocked HSP16F‐Cre and synHSPB′3‐Cre nucleofected cells at 42°C for either one 20‐min incubation or two 20‐min pulses (Figure S1A). As expected, the two 20‐min pulses produced higher GFP activation than the single 20‐min incubation. However, this was still lower than three 20‐min incubations. Under the two 20‐min condition, HSP16F cells showed an average of 8.1% GFP positivity, whereas synHSPB′3 reached 25.5%. As for thermal toxicity, the single 20‐min incubation at 42°C resulted in minimal cell loss, whereas the two 20‐min pulses at 42°C caused approximately 17% cell death across both HSPs (Figure S1B,C). There was no significant difference in cell loss between the two 20‐min and three 20‐min incubations. Overall, we concluded that three 20‐min heat shocks provide an optimal balance between robust GOI activation and acceptable cell viability.

### 2.4 Increasing the Genomic Integration Copies of synHSPB′3‐Cre and HSP16F‐Cre

**Figure 3:**
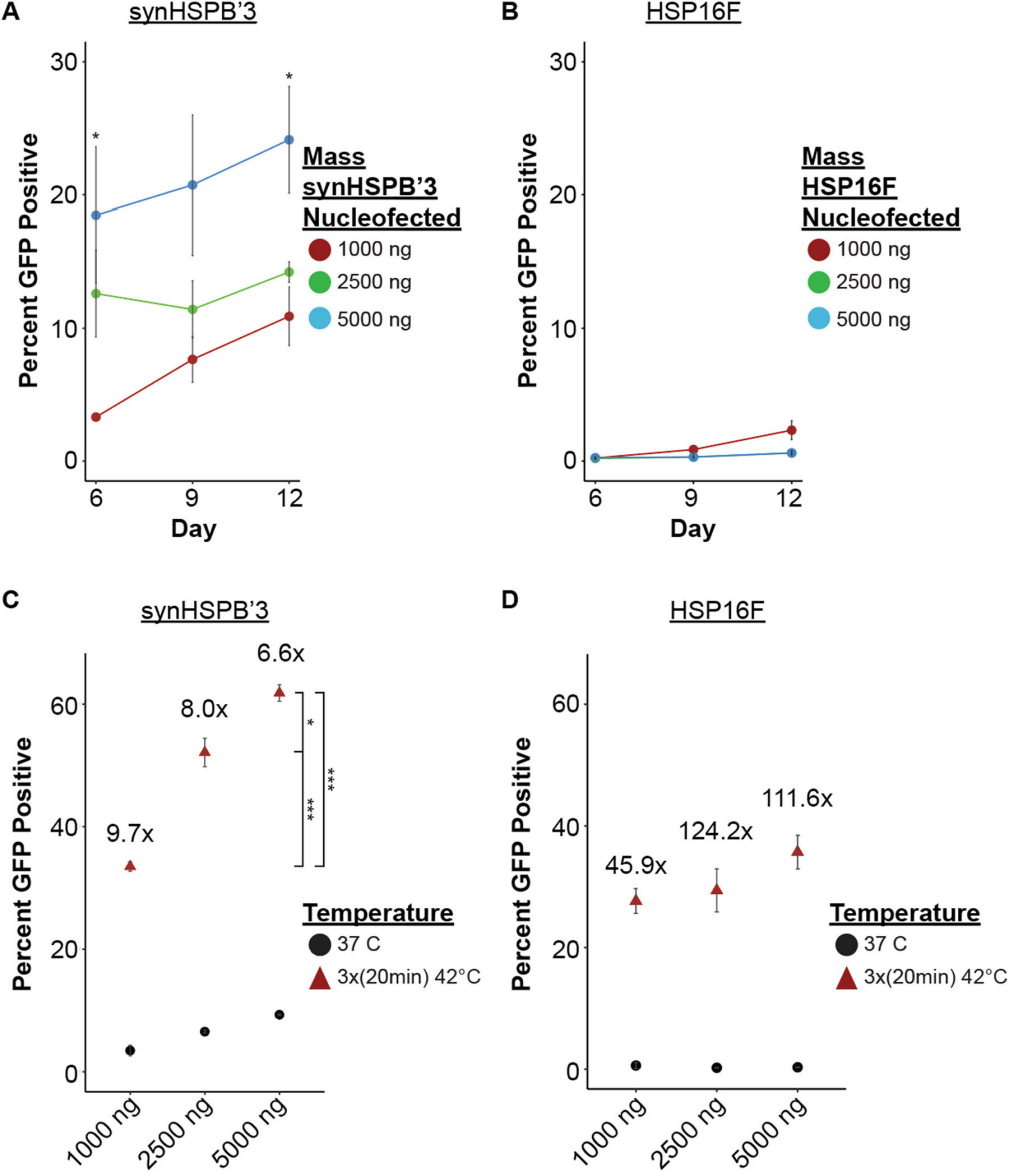
Effects on 37°C leakage and activation of synHSPB′3 and HSP16F by altering amount of genomic integration. (A and B) Time course 37°C leakage expression of GFP, with cells nucleofected on Day 0 with different amounts of the synHSPB′3‐Cre construct (A) or of the HSP16F‐Cre construct (B) (*n* = 3, one‐way ANOVA with post hoc Tukey test *p < 0.05). (C and D) Percent GFP positivity the day after heat shocking on Day 5, of cells nucleofected with different amounts of the synHSPB′3‐Cre construct (C) or of the HSP16F‐Cre construct (D). A total of 1000 ng data is the same from Figure 2B (*n* = 3, one‐way ANOVA with post hoc Tukey test *p < 0.05, ***p < 0.001).

Next, we evaluated the kinetics of leaky expression at 37°C beyond Day 6, with measurements taken on Days 9 and 12. Additionally, we assessed whether increased genomic integration led to higher leakage rates by comparing cells nucleofected with 1000 ng, 2500 ng, or 5000 ng of their respective HSP‐Cre transposons. As compared to the 1000 ng condition, synHSPB′3 exhibited significantly higher GFP expression for the cell nucleofected with 5000 ng on Days 6 and 12 (Figure 3A). In contrast, HSP16F showed no significant differences, with GFP expression consistently below 1% across all time points and masses tested (Figure 3B). These findings suggest that synHSPB′3 is more prone to 37°C leakage over time and with increased genomic integration, while HSP16F maintains tighter control.

Additionally, we investigated whether higher genomic integration, despite causing increased leaky expression with synHSPB′3, would proportionally enhance activation following non‐ultrasound‐induced heat shock, i.e. resulting in a higher fold change. The inverse, however, was observed for synHSPB′3 (Figure 3C). Although synHSPB′3 did not show proportional increases in activation relative to its leakage, it did exhibit a significant difference in the absolute GFP positivity following heat shock for all three tested masses (Figure 3C). For HSP16F, while the difference was not significant, the 5000 ng condition achieved 37% GFP positivity compared to ~28.5% for the 2500 and 1000 ng conditions (Figure 3D). These results indicate that synHSPB′3 produced the highest heat shock activation at 62%, whereas HSP16F demonstrated more stringent regulation at 37°C.

### 2.5 In Vitro FUS Mediated Heat Shock of synHSPB′3 and HSP16F

Finally, instead of using simple incubations, we applied FUS to heat shock the cells. Briefly, our setup involved suspending a 48‐well plate in a custom stand just at the surface of a 37°C degassed, deionized water bath to provide acoustic coupling. A 1.5 MHz focused transducer was submerged and mounted onto the well plate stand, centered and focused on the well containing the cells (Figure S2A,B), with signal sent from a waveform generator through an RF power amplifier. As a thermocouple could not be inserted during the experiment due to potential contamination of the cells, a preliminary sonication experiment was conducted to measure the temperature change. A dummy plate was prepared with an equivalent volume of cell media in the well of interest, and a thermocouple was inserted at the center of the well, almost touching the bottom. Sonication was performed for 20 min using a pulsed wave packet (100 ms burst period, 40% duty cycle), with the transducer's signal further pulsed on and off every 10 s. The temperature was measured using a digital thermometer with a Type K thermocouple (accuracy ±1.1°C). The heating profile increased gradually, reaching 43 ± 0.25°C at the center of the well after 6 min, and remained stable at this temperature for the remainder of the 20 min (Figure S2C). The approximate thermal dose equivalent heating at 43°C for 20 min of FUS sonication, as measured at the center of the well almost touching the bottom, was calculated to be 10.98 min. With the focus of the transducer properly aligned, the temperature measured at the edges of the well was 0.5 ± 0.2°C lower than measured in the center of the well. This experimental setup did not include a closed‐loop pressure feedback system to maintain temperature. The FUS protocol used was therefore chosen as it delivered a low thermal dose while maintaining a consistent temperature after a gradual rise. When cells are transferred between incubators, the temperature takes approximately 1 min to rise to 42°C or cool to 37°C. Although longer than the temperature rise time in the incubator, the gradual heating profile by FUS was chosen also to limit potential cell death and mechanical bioeffects possibly incurred at the higher intensity required for faster initial heating.

**Figure 4:**
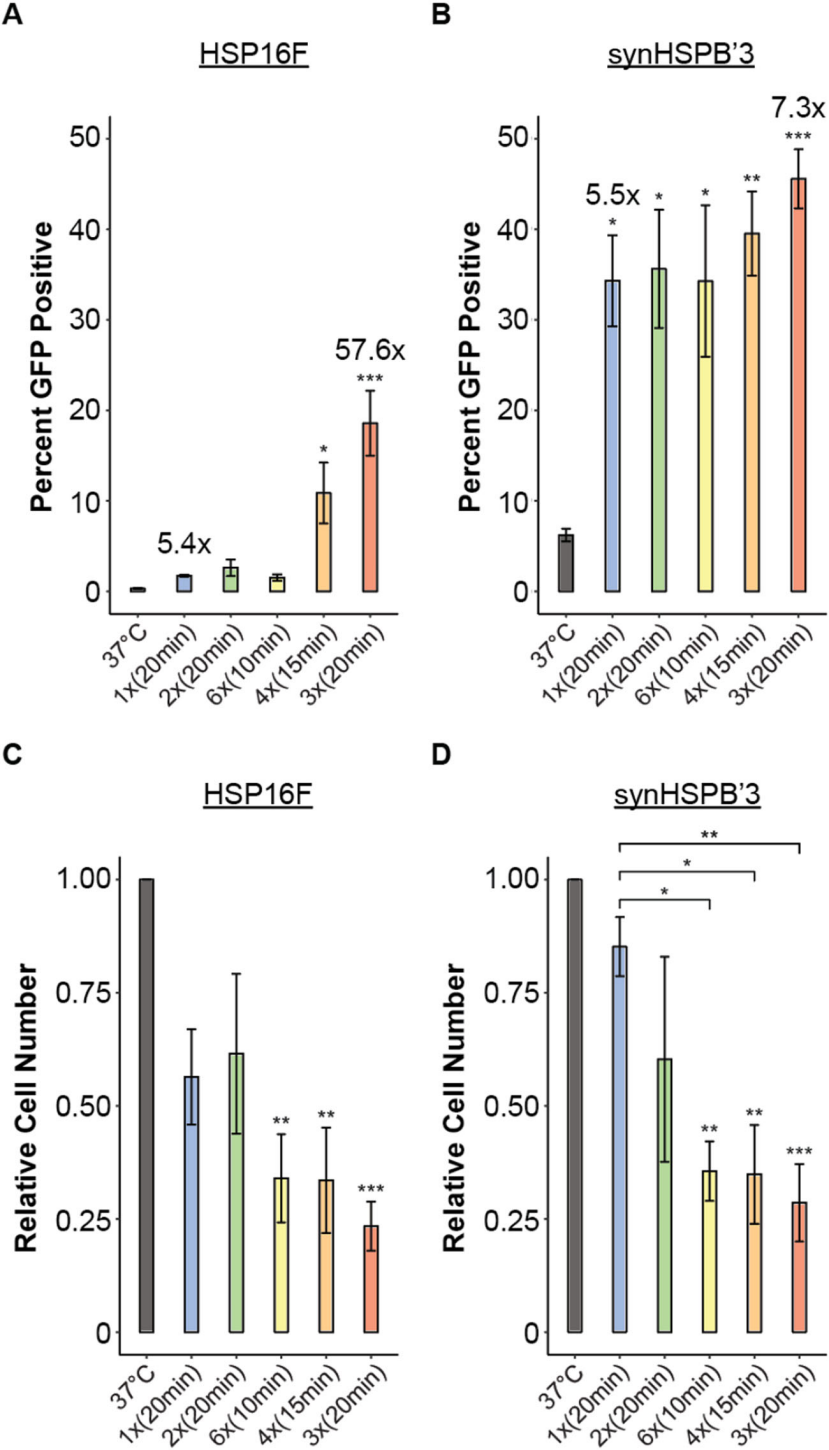
In vitro FUS heat shocking of HSP16F and synHSPB′3 hiPSCs. (A and B) Percent GFP positivity of cells nucleofected with either the HSP16F‐Cre (A) or the synHSPB′3‐Cre (B) construct across each FUS heat shock parameter. Points depict average and bars depict standard error (*n* = 3 for 1× (20 min) HSP16F, 2× (20 min) HSP16F, and 2× (20 min) synHSPB′3, *n* = 4 for rest, one‐way ANOVA with post hoc Tukey test *p < 0.05, **p < 0.01, ***p < 0.001). (C and D) Relative cell viability as a result of the pulsed FUS. Points depict average and bars depict standard error [*n* = 3 for 1× (20 min) HSP16F, 2× (20 min) HSP16F, and 2× (20 min) synHSPB′3, *n* = 4 for rest, one‐way ANOVA with post hoc Tukey test *p < 0.05, **p < 0.01, ***p < 0.001].

Using these FUS parameters, we heat shocked 1000 ng nucleofected HSP16F‐Cre and synHSPB′3‐Cre cells. Cells were subjected to FUS heat shock for either one 20‐min interval [1× (20 min)], two 20‐min pulsed intervals [2× (20 min)], or for a total of 1 h with cells sonicated at either 10, 15, or 20‐min pulsed intervals [6× (10 min), 4× (15 min), or 3× (20 min)] (Figure 4A,B). As expected, the 3× (20 min) FUS treatment resulted in the highest GFP positivity across the two HSP cell lines, with 18.6% for HSP16F and 45.6% for synHSPB′3. These were significant increases compared to their respective 37°C control groups of 0.3% and 6.2%, yielding fold changes of 57.6 and 7.3 (Figure 4A,B). Interestingly, for HSP16F, only the 4× (15 min) and 3× (20 min) FUS treatments resulted in a significant difference compared to its 37°C counterpart (Figure 4A). Whereas for synHSPB′3, all FUS conditions resulted in significantly higher GFP expression (Figure 4B). This indicates that synHSPB′3 requires a shorter duration of FUS heat shock than HSP16F to achieve activation. The cell loss for the 6× (10 min), 4× (15 min), and 3× (20 min) FUS treatments were significant compared to the 37°C control groups (Figure 4C,D). In addition, cells that received FUS for a total of 1 h [6× (10 min), 4× (15 min), and 3× (20 min)] were not significantly different from each other, averaging around 64%–77% cell death. These results demonstrate that FUS‐mediated hyperthermia effectively triggers the heat shock response, leading to significant GOI expression in hiPSCs by utilizing our HSP‐Cre/LoxP circuitry. However, sonication protocol must be carefully designed to minimize cell death.

## 3 Discussion

Inducible systems are critical tools in cutting‐edge biological research, allowing for precise spatiotemporal control of cellular processes (Ma et al. 2016; Chen et al. 2015; González et al. 2014; Reid et al. 2018; Kim et al. 2017; Yesbolatova et al. 2020). A relatively new and promising approach within this realm is to utilize FUS for non‐invasive actuation. In this study, we characterized the ability of six HSPs to induce gene expression in hiPSCs. By leveraging the cellular heat shock response, heat shock factors (HSFs) bind not only to their endogenous heat shock element loci but also to exogenous HSPs, which are integrated into the genome via transposase. Upon binding to the HSP, Cre expression is driven, leading to the excision of LoxP‐flanked DsRed and the subsequent constitutive expression of GFP or other GOIs. Our study used a single hiPSC line to establish feasibility. Because heat shock transcription factor (HSF1)‐mediated promoter activation is conserved, we anticipate broad transferability; however, line‑specific epigenetic contexts could modulate activation thresholds or maximal expression. Future studies will benchmark multiple hiPSC lines and primary cell types.

The main advantage of incorporating HSP‐Cre/LoxP circuitry is that a single heat shock treatment results in permanent GOI expression. If the GOI were placed directly downstream of the HSP, its expression would be relatively low and transient, primarily occurring during the heat shock. This is due to the rapid onset and termination of the heat shock response. However, the transient expression of Cre in our system is sufficient to induce robust and permanent GOI expression, enhancing the safety and feasibility of this design for in vivo applications. Using our HSP‐Cre/AAVS1‐LoxP‐DsRed‐STOP‐LoxP‐GFP system, we compared the performance of six different HSPs in hiPSCs. Cells were maintained at 37°C or non‐ultrasonically heat shocked at 42°C for 1 h via pulsed incubations. Of the six, synHSPB′3 and HSP16F performed best, likely due to their specific HSE sequences and the HSFs’ binding affinities to them.

For optimal performance, a balanced binding affinity is crucial. High binding affinity can lead to unwanted leakage at 37°C due to basal levels of activated HSFs even in non‐stressed states. This was observed in our testing of HSPB′1 and synHSPB′2. Conversely, low binding affinity results in low activation post‐heat shock, as observed with HSPmin, which showed very low GFP positivity. To further explore this, we tested whether increased genomic integration would proportionally alter the performance of HSP16F and synHSPB′3. Increasing the number of integrations raises the amount of substrate available for HSF binding. Interestingly, with HSP16F, no statistically significant differences were observed, likely due to saturation at 1000 ng. However, with synHSPB′3, significant differences in both 37°C leakage and heat shock activation were noted. These results suggest that our system, at least with synHSPB′3, is tunable. Depending on the desired balance between activation and leakage, the number of integrations can be adjusted accordingly.

Then we benchmarked our HSP‐Cre/LoxP system in hiPSCs via FUS, with parameters selected to balance the thermal dose needed for HSP activation while maintaining acceptable cell viability. Viabilities ranging from 23% to 85% were observed post‐sonication, and therefore, FUS parameters can be adjusted for in vivo applications to enhance viability while maintaining robust actuation. When comparing GFP positivity after 3× (20 min) FUS versus 3× (20 min) incubations, HSP16F performed worse with FUS, decreasing from 28% to 18.6% (Figures 2B and 4D). In contrast, synHSPB′3 performed better with FUS, increasing from 34% to 45.6% (Figures 2B and 4E). This contrast indicates that it is unlikely that mechanical stimulation by ultrasound waves engaged any cellular mechanisms which modulate the genetic expression resulting from the FUS heat shock. Additionally, comparing the one 20‐min FUS treatment to the 37°C control, HSP16F showed a modest increase in GFP positivity from 0.3% to 1.7%, while synHSPB′3 increased from 6.2% to 34.3% (Figure 4D,E). These findings suggest that synHSPB′3 is more sensitive to FUS and can achieve robust GOI expression at relatively lower thermal doses compared to HSP16F, resulting in safer treatment.

The advantage of using ultrasound waves for thermal stimulation lies in the ability to optimize key parameters—such as the waveform characteristics (e.g., frequency, pulse duration, duty cycle), beam geometry (e.g., focal depth and shape), and acoustic intensity—to non‐invasively deliver precise and controlled thermal doses to specific deep tissue targets. Utilizing real‐time temperature monitoring systems, heating protocols can be designed to meet the specific temperature and duration requirements necessary to activate inducible gene expression systems, while staying below the threshold of tissue damage. As therapeutic FUS applications continue to gain FDA approval, this technology will become increasingly accessible as a trigger for gene expression. hiPSCs have the unique ability to proliferate indefinitely and differentiate into specific somatic cells with high efficiency (Bao et al. 2016b; Lian et al. 2013, 2012, 2014, 2015; Bao 2016a; Jiang et al. 2021). This makes them ideal candidates not only for cell‐based regenerative therapy but also for coupling with FUS‐based inducible systems. In this study, we benchmarked an HSP‐Cre/LoxP circuitry in hiPSCs and demonstrated its robust ability to induce overexpression of GOIs through non‐ultrasound‐induced incubations and in vitro FUS. Future applications of this study could pave the way to synergistically enhance stem cell‐based regenerative therapies by inducing therapeutically relevant exogenous gene expression in situ via low intensity FUS.

## 4 Methods

### 4.1 Plasmid Cloning

All XLone‐HSP‐Cre plasmids were cloned into the XLone‐Cre general backbone. XLone‐Cre was cloned utilizing the In‐Fusion HD Cloning Kit (Takara, #639650, San Jose, CA, USA) according to the manufacture instructions. Briefly, XLone‐GFP (Addgene, #96930, Watertown, MA, USA) was restriction enzyme digested by KpnI and SpeI (New England Biolabs, #R3142S and #R3133S, respectively, Ipswich, MA, USA). The Cre insert was PCR amplified with Q5 High‐Fidelity 2X Master Mix (New England Biolabs, #M0492S, Ipswich, MA, USA) using the modRNAc1‐Cre template plasmid with primers designed by the Takara online In‐Fusion Cloning Primer Design Tool. Both the digested XLone‐GFP and PCR product were run through a 1% agarose gel. Expected sized bands were then excised and purified via the Zymoclean Gel DNA Recovery Kit (Zymo Research, #D4007, Irvine, CA, USA). The XLone backbone and the Cre PCR product were then In‐Fusion cloned using molar ratios calculated from the Takara online In‐Fusion molar ratio calculator, according to the manufacturer's instructions. Immediately after, half the reaction was transformed into Stbl3 Chemically Competent *E. coli* (Invitrogen, #C737303, Waltham, MA, USA), according to the manufacturer's protocol. A total of 200 µL were then plated onto Ampicillin agar selection plates and culture overnight at 37°C. Single colonies were then picked, cultured in 7.5 mL LB Broth (Gibco, # 10855001, Waltham, MA, USA) plus 100 µg/mL Ampicillin (Sigma‐Aldrich, #A5354, St. Louis, MO, USA) at 37°C, shaking at 250 rpm overnight, and miniprepped (Zymo Research, #D4211, Irvine, CA, USA) the following day. Clones were then whole plasmid sequenced to confirm sequence through Plasmidsaurus.

A successful XLone‐Cre plasmid was used to clone in each HSP. HSP sequences were generously provided to us from the Shapiro group. g‐blocks for each HSP were purchased through Genewiz (from Azenta Life Sciences). HSPs were then cloned into XLone‐Cre using the methods discussed above, however via restriction enzyme digestion with *Bst*XI and *Kpn*I (New England Biolabs, #R0113S and #R3133S, respectively, Ipswich, MA, USA). Successful clones were finally midiprepped (Invitrogen, #K210015, Waltham, MA, USA) for downstream experimentation.

### 4.2 Stem Cell Maintenance

The IMR90 hiPSC line was used for the duration of this study. They were maintained on iMatrix‐511 silk coated (IWAI, #N‐892021, Signal Hill, CA, USA) Falcon 6‐well plates (Corning, #353046, Corning, NY, USA), with daily complete media changes using essential 8 (E8) medium. Maintenance wells were passaged once confluency reached ~70%–80%. Briefly, cells were detached using Accutase (Innovative Cell Technologies, #AT104‐500, San Diego, CA, USA) and pelleted by centrifuging for 4 min at 200 rcf. Supernatant was then aspirated off and 1/12 the cells were re‐plated onto new iMatrix‐511 coated wells, that contained a final concentration of 5 µM Y27632 (Tocris, #1254, Minneapolis, MN, USA).

### 4.3 Generation of AAVS1‐LoxP‐DsRed‐STOP‐LoxP‐GFP Knockin Clonal Line

The AAVS1‐CAG‐LoxP‐DsRed‐STOP‐LoxP‐GFP donor plasmid was a gift from the Xiaoping Bao lab. IMR90 hiPSCs at about 70% confluency in a 6‐well plate were detached by treatment with Accutase and incubating the well at 37°C for 10 min. Cells were pelleted was resuspended in 100 µL PBS (Gibco, #14190136, Waltham, MA, USA) containing 7 µg donor plasmid, 7 µg of espCas9‐T2gRNA, and 2 µg pCE‐mp53DD (Addgene, #41856, Watertown, MA, USA). The T2 sgRNA (GGGGGCCACTAGGGACAGGAT) was ligated into the eSpCas9(1.1) plasmid (Addgene #71814, Watertown, MA, USA) that was digested with BbsI (New England Biolabs, #R0539S, Ipswich, MA, USA). The 100 µL cell suspension containing the DNA plasmids were then transferred into a cuvette and were nucleofected in a Lonza 4D Nucleofector, using the CB150 program. Cells were immediately transferred to 1 mL of pre‐warmed E8 media and recover for 10 min at 37°C. All the cells are then plated onto a new well of a 6‐well plate pre‐coated with iMartix‐511 and 5 µM ROCK inhibitor.

Cells were then expanded and gently drug selected in increasing concentrations of puromycin, to a final concentration of 1 µg/mL. This drug selected population was then sparsely single cell plated with 100 cells per well of a 6‐well plate. Once single cell colonies grew in size, media was changed with PBS containing 200 µg/mL Penicillin‐Streptomycin (Gibco, #15140122, Waltham, MA, USA) and were manually picked under a ×10 microscope. Cells manually picked from single cell colonies were immediately plated into individual wells of a 96‐well plate (Corning, #353072, Corning, NY, USA), precoated with iMatrix‐511, 5 µM ROCK inhibitor, and 200 µg/mL Penicillin‐Streptomycin. Clones were expanded then assessed for successful knock in by measuring DsRed positivity via flow cytometry.

### 4.4 Transposase Integration of XLone‐HSP‐Cre Constructs

Once the confluency of the reporter clonal line reached about 70%, cells were dissociated with Accutase for 10 min at 37°C, collected, and spun down. Cell pellet was resuspended in 100 µL PBS containing 1 µg of the respective XLone‐HSP‐Cre construct plasmid and 1 µg of the EF1α‐hyPBase plasmid. This 1:1 mass ratio between these two plasmids was maintained when 2.5 and 5 µg of the XLone‐HSP‐Cre construct was delivered. The DNA plasmid containing cell suspension was then transferred to a cuvette and nucleofected using the CB150 program on a Lonza 4D Nucleofector. Cells were immediately transferred to 1 mL of pre‐warmed E8 media and recovered for 10 min at 37°C. Cells were then plated onto new wells pre‐coated with iMartix‐511 and 5 µM ROCK inhibitor. For incubation heat shocking, cells were plated onto six well plates, and for FUS, they were plated onto 48 well plates (Corning, #353078, Corning, NY, USA). Daily media changes were conducted with 37°C pre‐warmed E8 media the following days, and were drug selected with a final concentration of 3 µg/mL blasticidin S (Sigma‐Aldrich, # 15205‐100MG, St. Louis, MO, USA).

### 4.5 Heat Shocking via 42°C Pulsed Incubations

The day of heat shocking, a secondary incubator was set to a temperature of 42°C and 5% CO_2_, at least 1 h prior. Additionally, 1 h prior, media for each well was changed with 37°C pre‐warmed E8 media containing 5 µM ROCK inhibitor. Once the incubator is at a stable temperature of 42°C, heat shock plates are quickly transferred from the 37°C incubator into the 42°C incubator. The plates remained in the 42°C incubator for either 20 min or 30 min. Once this time period was up, the plates were quickly transferred back into 37°C incubator and remained there for an equal amount of time. This cycle of transferring the plates was repeated until each plate experienced a total of 1 h at 42°C, upon which it remained at 37°C for the rest of the day. The next day cells were analyzed via flow cytometry.

### 4.6 Heat Shocking via FUS

The day of heat shocking, wells were changed with pre‐warmed E8 media with 5 µM ROCK inhibitor at least 1 h prior. Degassed, deionized water in a glass tank was kept at 37°C using a suis vide heater with precise temperature control (< 0.1°C) (Anova Precision Cooker Pro, Anova Culinary Inc., San Francisco, CA). As seen in Figure S2A,B, a well plate stand was designed to affix and align the transducer under the well of interest in a 48‐well plate holder. The stand consists of a 3D‐printed well plate holder with four screwed‐on metal legs. An adjustable metal arm with a 3D‐printed transducer holder attached at the end was secured onto one of the metal legs. A 1.5 MHz transducer (Blatek Industries Inc., Boalsburg, PA, USA) was then set into the transducer holder and centered below the well containing the cells. A small level and digital caliper were used to center and align the transducer at the focal distance. The degassed, deionized water was filled so that the bottom of the cell culture plate was submerged in the water to serve as impedance coupling. Each HSP cell line/FUS condition was plated on its own 48‐well plate, in the same well location, to easily switch out plates while maintaining the alignment of the transducer. To periodically ensure the alignment of the transducer between FUS conditions, an equivalent volume of cell media without cells was placed in the well of interest position in a dummy plate, and a test sine wave burst was sent to create an acoustic fountain that could visually be seen in the well of interest.

A sine wave burst (100 ms burst period, 40% duty cycle) was sent from a waveform generator (33250A 80 MHz Function/Arbitrary Waveform Generator, Agilent Technologies, Santa Clara, CA, USA) through a power amplifier (E&I 240 L linear RF Power Amplifier, 50 dB; Electronics & Innovation 115 Ltd., Rochester, NY, USA) to the transducer. The waveform generator was controlled by a MATLAB protocol to initiate the sonication protocol for 10 s, followed by a 10 s pause. This cycle was repeated in increments of 10 min, 15 min, or 20 min. Following the interval of sonication, each well plate was returned to a 37°C 5% CO_2_ control incubator for an equal interval of time. This process was repeated for the entire duration of the FUS treatment. The peak pressure in free‐space (water only) at the center of the focus was 1.03 MPa, measured using a needle hydrophone (HNR‐0500 Hydrophone‐preamplifier kit, ONDA Corporation, Sunnyvale, CA, USA). The in‐situ pressure field cannot be accurately measured due to standing waves formed in the presence of the well plate. Therefore, the in‐situ pressure field was numerically modeled using the COMSOL Multiphysics acoustic module. Frequency domain pressure acoustics using normal displacement of the transducer was used to calculate the pressure field. Air, water and acrylic plastic materials were selected from the COMSOL materials library using default temperature‐dependent material properties for speed of sound, density, dynamic viscosity, thermal conductivity, heat capacity and bulk viscosity. The global definitions for temperature were set to 310.15°K (37°C), and pressure to 1 atmosphere, to define the temperature‐dependent material properties. A maximum pressure of 1.68 MPa near the bottom and center of the well was estimated to be experienced by the cells. The free‐space pressure field was also modeled and is shown in Figure S3A for comparison. Default material properties are also elaborated in Figure S3A.

To prevent contamination of the cell environment, a temperature probe could not be inserted to measure the temperature of the cells directly during heat shock. Instead, a dummy plate filled with 500 µL of pre‐warmed E8 media without cells was used to calibrate the system before each experiment. A temperature probe was placed nearly touching the center of the bottom of the well, enclosed and secured by putty covering the filled well in the dummy plate. After transducer focus and alignment were checked to confirm no fountaining effects within the well, a 20‐min heat profile was gathered to estimate thermal dose during the intervals of hyperthermia by FUS (Figure S2C).

### 4.7 Live Cell Flow Cytometry Analysis

For analysis of DsRed and GFP expression, we performed live cell flow cytometry. Cells were washed with PBS to removed debris and detached cells. Then, remaining cells were treated with Accutase for 7 min at 37°C and pelleted by centrifuging for 4 min at 200 rcf. Supernatant was then aspirated off and cells were resuspended in PBS + 5% fetal bovine serum (VWR, #97068‐085, Radnor, PA, USA) + 2% Penicillin Streptomycin (Corning, #30002Cl, Corning, NY, USA). Cells were immediately run through a BD Accuri C6 Plus flow cytometer and the flow data was analyzed on FlowJo.

## Author Contributions

**Alessandro R. Howells:** plasmid cloning, cell culture work, flow cytometry, data analysis, and wrote manuscript. **Tahir Haideri:** establish the knockin clone and perform the differentiation experiment. **Kama Bell** and **Hyeonu Heo:** focused ultrasound and wrote manuscript. **Xiaojun Lance Lian** and **Yun Jing:** wrote manuscript and supervised project.

## Conflicts of Interest

The authors declare no conflicts of interest.

## Supporting information

Supp figures. **Figure S1:** HSP16F and synHSPB'3 1x and 2x 20‐minute 42°C incubation heat shocking. A) Percent GFP positivity across the two HSPs and across each heat shock parameter. Points depict averages and bars depict standard error. (*n* = 3, one‐way ANOVA with post‐hoc Tukey test *p < 0.05, **p < 0.01, ***p < 0.001). Fold change increase shown is calculated using the averages from the 2x(20min) group and the 37°C control group. B and C) Relative cell viability as a result of the pulsed 42°C incubations for HSP16F (B) and synHSPB'3 (C) cells. Points depict average and bars depict standard error (*n* = 3). Note: 3x(20min) data is the same from Figure 2C. **Figure S2:** In vitro FUS set up. A and B) Photographs of our FUS setup. A heater is used to hold a tank of degassed, deionized water at 37°C. A single element focused ultrasound transducer with a center frequency of 1.5 MHz is attached to a 3D printed well plate stand via an adjustable arm and aligned using a level and digital caliper. The transducer is centered underneath the well containing cells in a 48 well plate. Each HSP cell line/FUS condition was plated on its own 48‐ well plate, in the same well location, to easily switch out plates without disrupting the alignment of the transducer. Pulsed FUS is applied with an amplitude of 1 MPa, 100 ms burst period and 40% duty cycle for 10 seconds, following 10 seconds of rest, repeated for an increment of 10, 15 or 20 minutes. A dummy plate with equivalent volume of cell medium was used to periodically check the alignment of the transducer via acoustic fountain. C) Temperature rise in Celsius due to FUS hyperthermia, measured in cell‐less culture medium using a digital temperature probe and type K thermocouple to estimate heat shocking experienced by cells during the longest sonication duration. With the transducer properly centered, temperature at the center of the focal point was measured to be 0.5±0.1°C higher than at the edges of the well of interest. **Figure S3:** Numerically modeled FUS pressure fields. COMSOL simulation using frequency domain pressure acoustics normal displacement to calculate the pressure field. Default material properties for water, acrylic plastic and air were used by choosing the materials from COMSOL's materials library. The global definitions for pressure and temperature were set to 1 atmosphere and 310.15 K, as in the experimental control incubator to define the default temperaturedependent material properties. A) Free field pressure in water, with the transducer represented by the curvature at the bottom of the image. The maximum pressure is 1.03 MPa in this case. B) Pressure field with the presence of a well plate. A layer of acrylic plastic is used to represent the well bottom, with air above the acrylic plastic layer and above the water volume inside the well. The presence of the acrylic plastic layer and air pocket in the well create standing waves with a maximum pressure of 1.68 MPa near the bottom of the well.

## Data Availability

The data that support the findings of this study are available from the corresponding author upon reasonable request.
