# Supplementary material for: Ultrasound Control of Gene Expression in Human iPSCs via Heat Shock Promoters
Source: Biotechnol Bioeng. 2025 Aug 20;122(11):3216–28. doi: 10.1002/bit.70050 (PMC12503016; doi:10.1002/bit.70050)
Supplement: Supplementary file 1 — Supp figures. Figure S1: HSP16F and synHSPB'3 1x and 2x 20‐minute 42°C incubation heat shocking. A) Percent GFP positivity across the two HSPs and across each heat shock parameter. Points depict averages and bars depict standard error. (n = 3, one‐way ANOVA with post‐hoc Tukey test *p < 0.05, **p < 0.01, ***p < 0.001). Fold change increase shown is calculated using the averages from the 2x(20min) group and the 37°C control group. B and C) Relative cell viability as a result of the pulsed 42°C incubations for HSP16F (B) and synHSPB'3 (C) cells. Points depict average and bars depict standard error (n = 3). Note: 3x(20min) data is the same from Figure 2C. Figure S2: In vitro FUS set up. A and B) Photographs of our FUS setup. A heater is used to hold a tank of degassed, deionized water at 37°C. A single element focused ultrasound transducer with a center frequency of 1.5 MHz is attached to a 3D printed well plate stand via an adjustable arm and aligned using a level and digital caliper. The transducer is centered underneath the well containing cells in a 48 well plate. Each HSP cell line/FUS condition was plated on its own 48‐ well plate, in the same well location, to easily switch out plates without disrupting the alignment of the transducer. Pulsed FUS is applied with an amplitude of 1 MPa, 100 ms burst period and 40% duty cycle for 10 seconds, following 10 seconds of rest, repeated for an increment of 10, 15 or 20 minutes. A dummy plate with equivalent volume of cell medium was used to periodically check the alignment of the transducer via acoustic fountain. C) Temperature rise in Celsius due to FUS hyperthermia, measured in cell‐less culture medium using a digital temperature probe and type K thermocouple to estimate heat shocking experienced by cells during the longest sonication duration. With the transducer properly centered, temperature at the center of the focal point was measured to be 0.5±0.1°C higher than at the edges of the well of interest. Figure S3: Nume [file BIT-122-3216-s001.pdf]

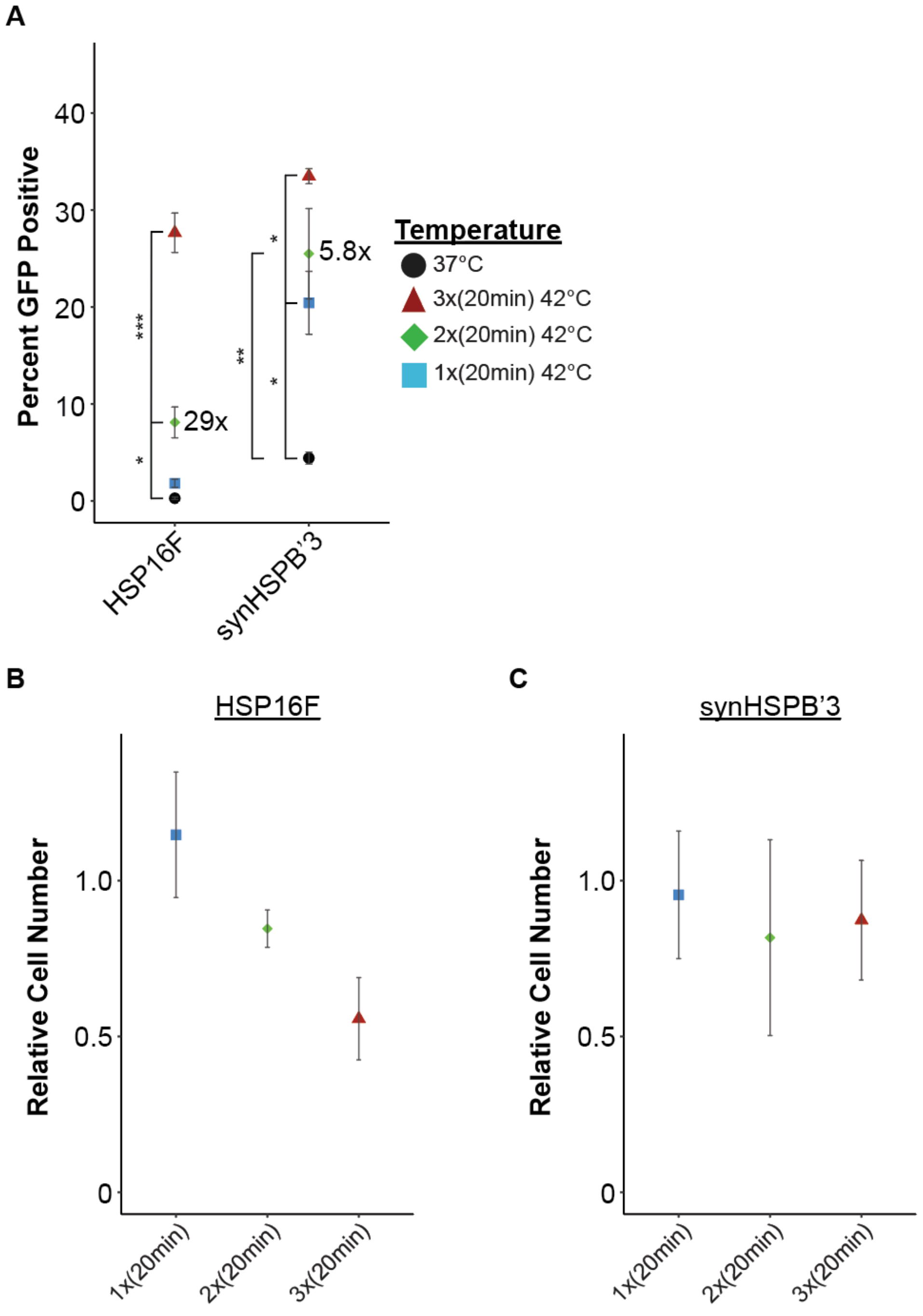

Figure S1: HSP16F and synHSPB'3 1x and 2x 20-minute 42 °C incubation heat shocking. A) Percent GFP positivity across the two HSPs and across each heat shock parameter. Points depict averages and bars depict standard error. (n=3, one-way ANOVA with post-hoc Tukey test *p < 0.05, **p < 0.01, ***p < 0.001). Fold change increase shown is calculated using the averages from the 2x(20min) group and the 37°C control group. B and C) Relative cell viability as a result of the pulsed 42°C incubations for HSP16F (B) and synHSPB'3 (C) cells. Points depict average and bars depict standard error (n=3). Note: 3x(20min) data is the same from Figure 2C.

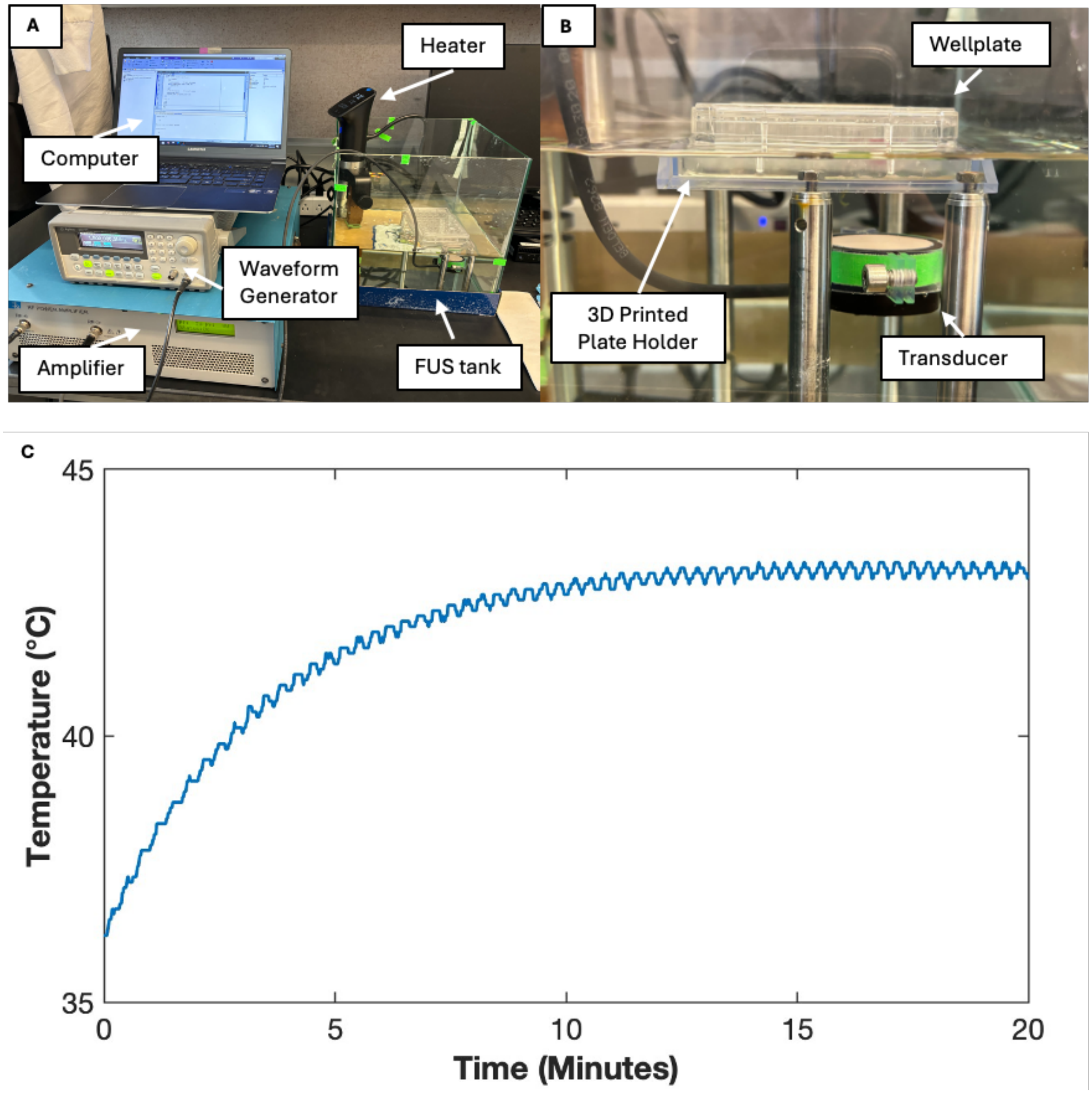

Figure S2: In vitro FUS set up. A and B) Photographs of our FUS setup. A heater is used to hold a tank of degassed, deionized water at 37°C. A single element focused ultrasound transducer with a center frequency of 1.5 MHz is attached to a 3D printed well plate stand via an adjustable arm and aligned using a level and digital caliper. The transducer is centered underneath the well containing cells in a 48 well plate. Each HSP cell line/FUS condition was plated on its own 48-well plate, in the same well location, to easily switch out plates without disrupting the alignment of the transducer. Pulsed FUS is applied with an amplitude of 1 MPa, 100 ms burst period and 40% duty cycle for 10 seconds, following 10 seconds of rest, repeated for an increment of 10, 15 or 20 minutes. A dummy plate with equivalent volume of cell medium was used to periodically check the alignment of the transducer via acoustic fountain. C) Temperature rise in Celsius due to FUS hyperthermia, measured in cell-less culture medium using a digital temperature probe and type K thermocouple to estimate heat shocking experienced by cells during the longest sonication duration. With the transducer properly centered, temperature at the center of the focal point was measured to be 0.5±0.1°C higher than at the edges of the well of interest.

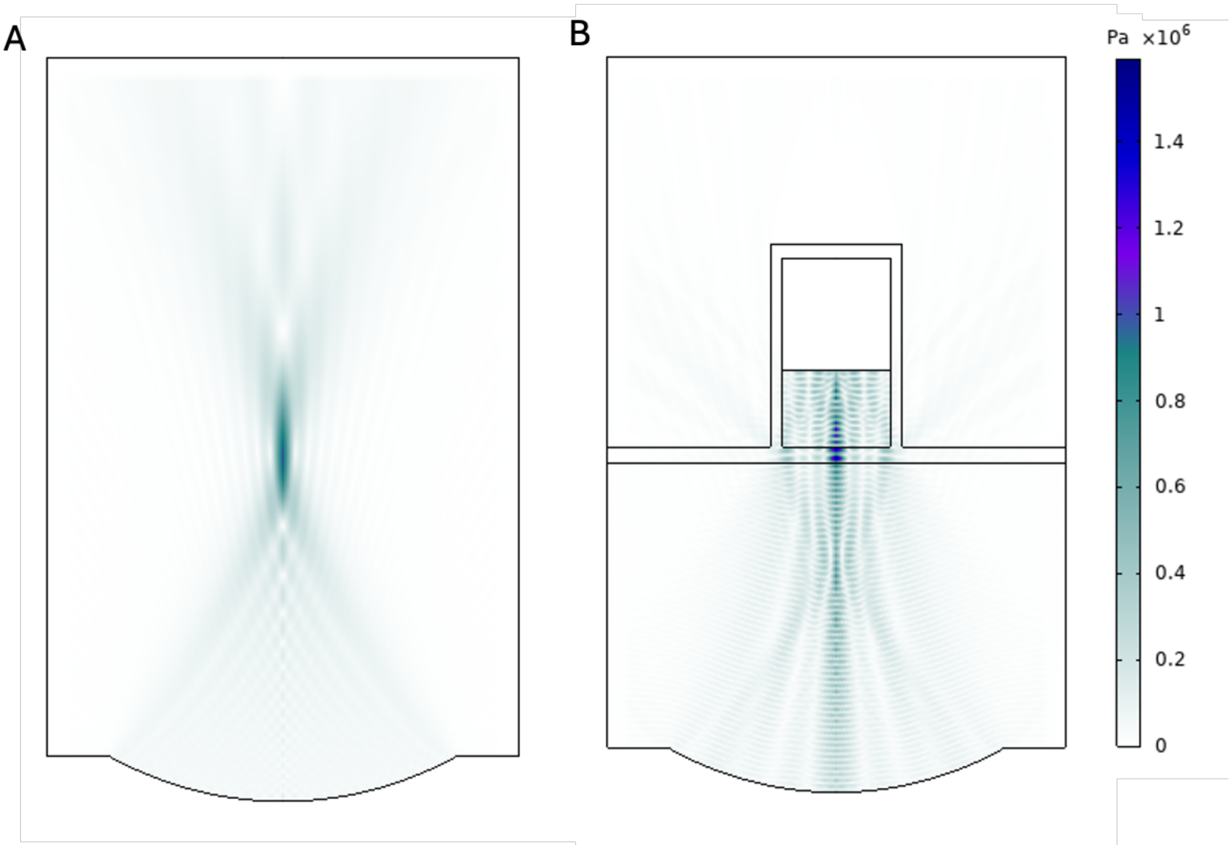

Figure S3: Numerically modeled FUS pressure fields. COMSOL simulation using frequency domain pressure acoustics normal displacement to calculate the pressure field. Default material properties for water, acrylic plastic and air were used by choosing the materials from COMSOL's materials library. The global definitions for pressure and temperature were set to 1 atmosphere and 310.15 K, as in the experimental control incubator to define the default temperature-dependent material properties. A) Free field pressure in water, with the transducer represented by the curvature at the bottom of the image. The maximum pressure is 1.03 MPa in this case. B) Pressure field with the presence of a well plate. A layer of acrylic plastic is used to represent the well bottom, with air above the acrylic plastic layer and above the water volume inside the well. The presence of the acrylic plastic layer and air pocket in the well create standing waves with a maximum pressure of 1.68 MPa near the bottom of the well.
